# Integrated Analysis of ceRNA Network Reveals Prognostic and Metastasis Associated Biomarkers in Breast Cancer

**DOI:** 10.3389/fonc.2021.670138

**Published:** 2021-05-13

**Authors:** Da Qian, Qinghui Zheng, Danping Wu, Buyun Ye, Yangyang Qian, Tao Zhou, Jie Qiu, Xuli Meng

**Affiliations:** ^1^College of Medicine, Soochow University, Soochow, China; ^2^Department of Breast Surgery, Zhejiang Provincial People’s Hospital, Hangzhou, China; ^3^Department of Burn and Plastic Surgery-Hand Surgery, First People’s Hospital of Changshu City, Changshu Hospital Affiliated to Soochow University, Soochow, China; ^4^Department of Breast Surgery, First People’s Hospital of Changshu City, Changshu Hospital Affiliated to Soochow University, Soochow, China; ^5^Second Clinical College, Zhejiang Chinese Medical University, Hangzhou, China; ^6^Faculty of Basic Medicine, Hangzhou Medical College, Hangzhou, China

**Keywords:** breast cancer, metastasis, ceRNA network, biomarkers, prognostic

## Abstract

**Background:**

Breast cancer is a malignancy and lethal tumor in women. Metastasis of breast cancer is one of the causes of poor prognosis. Increasing evidences have suggested that the competing endogenous RNAs (ceRNAs) were associated with the metastasis of breast cancer. Nonetheless, potential roles of ceRNAs in regulating the metastasis of breast cancer remain unclear.

**Methods:**

The RNA expression (3 levels) and follow-up data of breast cancer and noncancerous tissue samples were downloaded from the Cancer Genome Atlas (TCGA). Differentially expressed and metastasis associated RNAs were identified for functional analysis and constructing the metastasis associated ceRNA network by comprehensively bioinformatic analysis. The Kaplan-Meier (K-M) survival curve was utilized to screen the prognostic RNAs in metastasis associated ceRNA network. Moreover, we further identified the metastasis associated biomarkers with operating characteristic (ROC) curve. Ultimately, the data of Cancer Cell Line Encyclopedia (CCLE, https://portals.broadinstitute.org/ccle) website were selected to obtained the reliable metastasis associated biomarkers.

**Results:**

1005 mRNAs, 22 miRNAs and 164 lncRNAs were screened as differentially expressed and metastasis associated RNAs. The results of GO function and KEGG pathway enrichment analysis showed that these RNAs are mainly associated with the metabolic processes and stress responses. Next, a metastasis associated ceRNA (including 104 mRNAs, 19 miRNAs, and 16 lncRNAs) network was established, and 12 RNAs were found to be related to the overall survival (OS) of patients. In addition, 3 RNAs (hsa-miR-105-5p, BCAR1, and PANX2) were identified to serve as reliable metastasis associated biomarkers. Eventually, the results of mechanism analysis suggested that BCAR1 might promote the metastasis of breast cancer by facilitating Rap 1 signaling pathway.

**Conclusion:**

In the present research, we identified 3 RNAs (hsa-miR-105-5p, BCAR1 and PANX2) might associated with prognosis and metastasis of breast cancer, which might be provide a new perspective for metastasis of breast cancer and contributed to the treatment of breast cancer.

## Introduction

Breast cancer, one of the most common malignant tumors in females, is highly invasive and metastatic (1). Breast is not an important organ to maintain human life activities and breast cancer *in situ* is not fatal. However, as breast cancer cells lose the characteristics of normal breast cells, the connection between them become loose and easy to fall off (2). Once the cancer cells fall off, the free cancer cells can spread throughout the body with blood or lymph, forming lung, brain, bone and liver metastasis. The median survival of breast cancer patients with metastasis is only 18 to 24 months (3–6). Although mammographic screening may lower the metastasis-related mortality, the method is inappropriate for detection at early stages (7). Therefore, it is urgent to screen novel biomarkers to predict the prognosis and monitor metastasis of breast cancer and further explore the potential mechanisms.

Competing endogenous RNAs (ceRNAs) reveal a new mechanism of RNAs interaction (8). MicroRNAs (miRNAs) can cause gene silencing by binding to mRNAs, while lncRNAs can regulate the expression of the target genes by competitively binding to miRNAs (9, 10). Moreover, lncRNAs also have been reported to play a significant role in embryonic and cancer development (11–14). In addition, studies have suggested that the abnormal expression of ceRNAs is closely related to the occurrence, development and prognosis of tumors, including breast cancer (15). For example, Fan et al. identified 4 lncRNAs as independent prognostic biomarkers of breast cancer patients by constructing a ceRNA network (16). On the other hand, Yao et al. also provided novel insights into the drugs treatment and prognosis of breast cancer patients from ceRNA network (17). However, the research of ceRNA network on metastasis of breast cancer is not comprehensive.

The present study aims to construct a metastatic ceRNA network of breast cancer based on a series of bioinformatic analysis. Moreover, prognostic and metastatic biomarkers were identified by Kaplan-Meier (K-M) survival analysis and receiver operating characteristic (ROC) curves, respectively. The constructed ceRNA and identified biomarkers can not only contribute to further understand the molecular mechanism of metastatic breast cancer, but also improve clinical diagnosis the metastasis of breast cancer, which may contribute to the treatment of metastatic breast cancer.

## Materials and Methods

### Data Collection

The RNAs expression profiles (mRNAs, miRNAs, and lncRNAs) including fragments per kilobase of exon per million reads mapped (FPKM) and follow-up data of 899 primary breast cancer patients (including 878 M0 patients and 21 M1 patients, and all of the tissue samples from the primary site) and 90 noncancerous tissue samples (including 86 paired paracancerous M0 tissue samples, 1 paired paracancerous M1 tissue samples and 3 unpaired normal tissue samples) were downloaded from the Cancer Genome Atlas (TCGA, https://portal.gdc.cancer.gov/) database. The breast cancer patients without the information of M0 stage or M1 stage and were excluded.

### Identification of Differentially Expressed and Metastasis-Associated RNAs

When the expression of RNAs (including lncRNAs, miRNAs, and mRNAs) were detected in both cancer patients and noncancerous tissue samples, the RNAs were preserved for further analysis. The Limma package in R was utilized to screen the differentially expressed RNAs (DERs, including DElncRNAs, DEmiRNAs, and DEmRNAs) between 899 primary breast cancer patients and 90 noncancerous tissue samples (18). The p<0.05 was considered the screening standard. Then, the ggplot2 v.3.3.2 package in R was selected and visualized the volcano plots of DERs. Similarly, metastasis associated RNAs (MAR, including MAlncRNAs, MAmiRNAs, and MAmRNAs) which were differentially expressed between 878 M0 patients and 21 M1 patients were identified with the Limma package in R. Finally, overlapping RNAs between DERs and MARs which were defined as differentially expressed and metastasis associated RNAs (DEMARs, including DEMAlncRNAs, DEMAmiRNAs, DEMAmRNAs) were extracted for subsequent analysis and visualized by a venn diagram.

### Enrichment Analysis

WEBGASTALT (http://www.webgestalt.org/webgestalt_2013/) website was used to investigate the biological function of overlapping DEMAR (19). The Gene Ontology (GO) annotation and the Kyoto Encyclopedia of Genes and Genomes (KEGG) pathways were selected to explore the biological function of DEMAmRNA. Moreover, the biological function of the DEMAlncRNA and DEMAmiRNAs were investigated by the DIANA miRPath v.3 web-based computational tool (http://snf515788.vm.okeanos.grnet.gr/) (20). When the terms of GO (including biological processes, molecular function and cellular component) and KEGG showed p<0.05, theses terms were considered significantly enriched.

### Construction of the Metastasis Associated ceRNA Network

DEMAlncRNAs, DEMAmiRNAs, DEMAmRNAs were used to construct the metastasis associated ceRNA network. Firstly, we predicted the target genes of DEMAmiRNAs by miranda 3.3a software (21). Second, common genes between the predictive target genes and the DEMAmRNAs were retained for analysis. Thirdly, Pearson’s correlation analysis was performed to identified the mRNAs which were negatively regulated by DEMAmiRNAs by psych v.2.0.12 package in R. Moreover, we also obtained the negatively correlated interactions between DEMAlncRNAs and DEMAmiRNAs by miranda 3.3a software and Pearson’s correlation analysis. Finally, Cytoscape v.3.7.2 was used to constructed a metastasis associated ceRNA network based on the negatively correlated interactions between DEMAmiRNAs and DEMAmRNAs and between DEMAmiRNAs and DEMAlncRNAs (22).

### Survival Analysis

The Kaplan-Meier (K-M) survival curve was drawn to explore the relationship between the expression levels of RNAs in metastasis associated ceRNA network and the overall survival (OS) of breast cancer patients by survminer v.0.4.6 package in R (23). All breast cancer patients were split into high expression and low expression based on the median expression of each RNA in metastasis associated ceRNA network. Then, Kaplan-Meier survival curves were used to assess the prognostic value of each RNA. RNAs which were associated with the overall survival (P<0.05) were retained for further analysis.

### Identification and Validation of the Metastatic Biomarkers

The receiver operating characteristic (ROC) curve was plotted to identify the metastatic biomarkers and area under the ROC curve (AUC) was calculated using pROC v.1.16.2 package in R to show the performance of distinguishing M0 and M1 samples (24). Next, we screened the easily metastatic cell lines and not easily metastatic cell lines form the Cancer Cell Line Encyclopedia (CCLE, https://portals.broadinstitute.org/ccle) website to detect the expression level of metastatic biomarkers. Moreover, we further validate the expression levels of each metastatic biomarkers between 878 M0 and 21 M1 samples from the TCGA database employing Wilcoxon rank sum test. P< 0.05 was set as a statistically significant difference. Furthermore, we also compared the expression levels of each metastatic biomarkers between 87 cancer samples and paired 87 paired paracancerous tissue samples.

### Mechanism Analysis of Metastatic Biomarkers

To better elucidate the regulatory mechanism of metastatic biomarkers, we extracted the regulatory network that was associated to the metastatic biomarkers and plotted Sankey diagram to show the regulatory mechanism of each metastatic biomarkers using ggalluvial v.0.12.3 package in R. Moreover, we also extracted the cancer associated KEGG pathways and drawn a graph to show the regulatory mechanism of metastatic biomarkers with Adobe Illustrator (Adobe Systems, Mountain View, CA, USA).

### Statistical Analysis

All statistical analyses were accomplished by R Studio (R Version 4.0.2) software. The log-rank test was used in the Kaplan-Meier survival curve analysis. P-value <.05 was set as the standard of statistical analysis.

## Result

### Identification of Differentially Expressed and Metastasis-Associated RNAs

**Figure 1:**
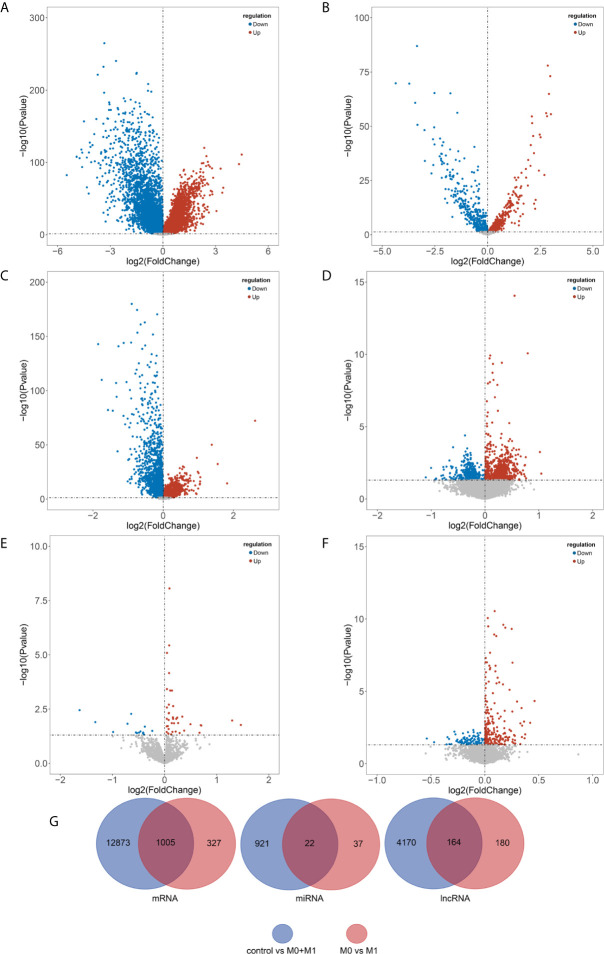
Identification of differentially expressed and metastasis associated RNAs. DEmRNAs **(A)**, DEmiRNAs **(B)**, and DElncRNAs **(C)** between breast cancer patients and noncancerous tissue samples and MAmRNAs **(D)**, MAmiRNAs **(E)**, and MAlncRNAs **(F)** between M0 and M1 samples were presented using volcano plots. Venn diagram showing the overlapped the DERs and MARs **(G)**. Blue, downregulated; red, upregulated; black, not differential expressed. DEmRNAs, differentially expressed mRNAs; DEmiRNAs, differentially expressed miRNAs; DElncRNAs, differentially expressed lncRNAs; MAmRNAs, metastasis associated mRNAs; MAmiRNAs, metastasis associated miRNAs; MAlncRNAs, metastasis associated lncRNAs.

In the present study, we used Limma package in R to screen the DERs and MARs. Under the cut-off of P-value < 0.05, a total of 13,878 DEmRNAs (including 7,198 upregulated and 6,680 downregulated), 943 DEmiRNAs (including 483 upregulated and 460 downregulated) and 4,334 DElncRNAs (including 1,706 upregulated and 2,628 downregulated) were identified between breast cancer patients and noncancerous tissue samples (**Supplementary Tables S1**–**S3** and **Supplementary Figure S1A**). Moreover, a total of 1332 MAmRNAs (including 798 upregulated and 534 downregulated), 59 MAmiRNAs (including 45 upregulated and 14 downregulated) and 344 MAlncRNAs (including 98 upregulated and 246 downregulated) were identified between M0 and M1 samples (**Supplementary Tables S4**–**S6** and **Supplementary Figure S1B**). Volcano plots were plotted to present the DERs and MARs, as shown in **Figures 1A–F**. Furthermore, after obtaining the intersection of DERs and MARs, 1005 mRNAs, 22 miRNAs and 164 lncRNAs were obtained as DEMARs (**Figure 1G**).

### Enrichment Analysis

**Figure 2:**
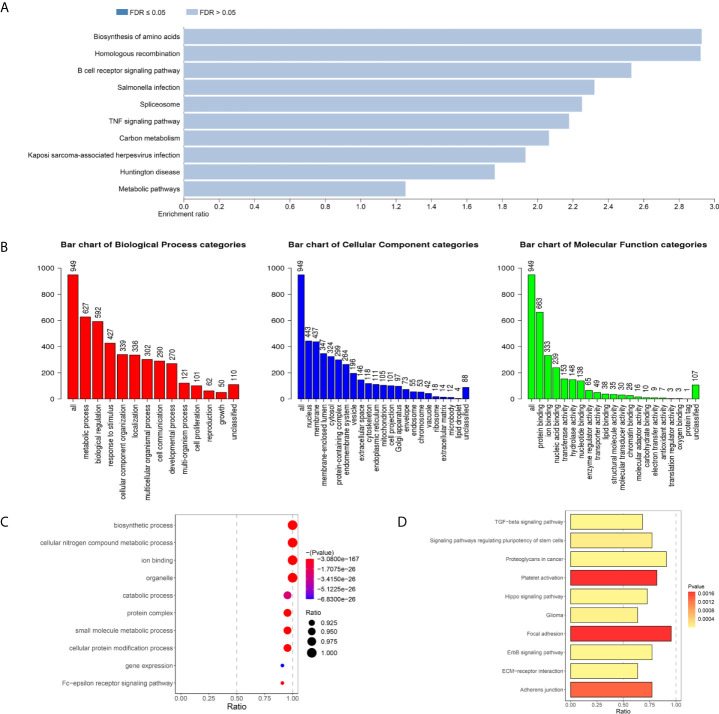
The biological function of (KEGG pathways (P < 0.05) of DEMAmRNAs **(A)** and DEMAmiRNAs **(D)**. GO terms (P < 0.05) of DEMAmRNAs **(B)** and DEMAmiRNAs **(C)**, KEGG, Kyoto Encyclopedia of Genes and Genomes; GO, Gene Ontology; DEMARs, differentially expressed and metastasis associated RNAs; DEMAmRNAs, differentially expressed and metastasis associated mRNA; DEMAmiRNAs, differentially expressed and metastasis associated miRNAs.

To further investigate the biological function of DEMARs in the breast cancer metastasis, GO function and KEGG pathway enrichment analysis were performed using WEBGASTALT website for differentially expressed and metastasis associated mRNAs. The results of KEGG pathway analysis showed DEMAmRNAs mainly regulate the biosynthesis of amino acids, homologous recombination, B cell receptor signaling pathway, salmonella infection, spliceosome, TNF signaling pathway, Carbon metabolism, and so on (**Supplementary Table S7** and **Figure 2A**). Besides, as shown in **Figure 2B**, the result of GO analysis suggested that DEMAmRNAs mainly involved in metabolic processes and stress responses (biological process), nucleus and membrane (Cellular component), and protein binding and ion binding (molecular function, **Supplementary Table S8**). Furthermore, we also analyzed the biological function of DEMAlncRNAs and DEMAmiRNAs by miRPath v.3 web-based computational tool, and the results also suggested that DEMAmiRNAs primarily related to biosynthetic process, cellular nitrogen compound metabolic process, ion binding, TGF-beta signaling pathway, Hippo signaling pathway, Wnt signaling pathway, PI3K-Akt signaling pathway, RAP1 signaling pathway and RAS signaling pathway (**Supplementary Tables S9**, **S10** and **Figures 2C, D** **)**. However, DEMAlncRNAs mainly associated with the detection of chemical stimulus involved in sensory perception of smell and detection of chemical stimulus involved in sensory perception (**Supplementary Figure S2**). In short, the results of enrichment analysis implied that the DEMARs may play an important role for breast cancer metastasis by regulating above pathways or biological processes.

### Construction of the Metastasis Associated ceRNA Network

In order to construct a metastasis associated ceRNA network, we firstly predicted the target genes of 22 DEMAmiRNAs and obtained the intersection of target genes and DEMAmRNAs. Next, Pearson’s correlation analysis was performed to identified the mRNAs which were negatively regulated by DEMAmiRNAs. Finally, negatively regulated miRNAs and mRNAs were preserved to build ceRNA network. Similarly, negatively regulated miRNAs and lncRNAs were preserved to build ceRNA network.

**Figure 3:**
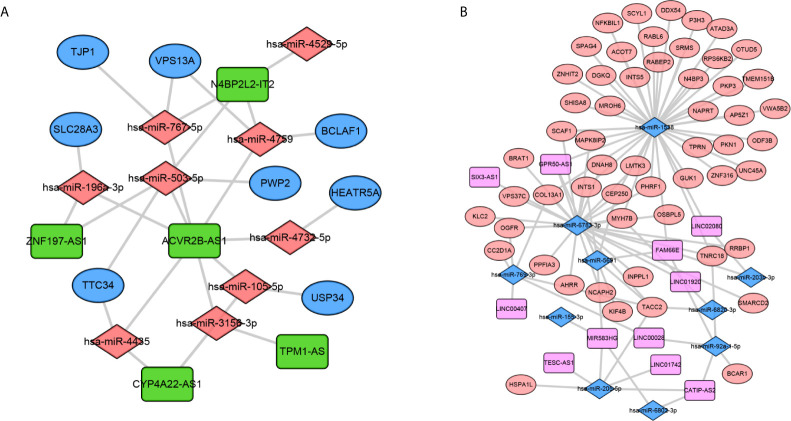
Construction of the metastasis associated ceRNA networks. Upregulated miRNAs ceRNA network **(A)** and downregulated miRNAs ceRNA network **(B)**. In network A, orange diamond: upregulated miRNAs, green rectangle: downregulated lncRNAs, Blue Oval: downregulated mRNAs. In network B, orange oval: upregulated mRNAs, pink rectangle: upregulated lncRNAs, blue diamond: downregulated miRNAs.

Based on the negatively regulated interaction pairs between DEMAmiRNAs and DEMAmRNAs and between DEMAmiRNAs and DEMAlncRNAs, a metastasis associated ceRNA network with 139 differentially expressed and metastasis associated RNAs (including 104 mRNAs, 19 miRNAs, and 16 lncRNAs) was established (**Tables 1**–**3** and **Figure 3**). In addition, to directly display the expression level of DEMAmiRNAs, two ceRNA sub-networks including upregulated miRNAs in M1 samples and downregulated miRNAs in M1 samples were plotted by Cytoscape v.3.7.2 (**Figures 3A, B** **)**.

**Table 1:** Differentially expressed and metastasis associated mRNAs in ceRNA network.

	logFC	P Value	adj.P.Val
PPFIA3	0.479089697	0.002372667	0.344801451
ACOT7	0.477983617	0.002708625	0.366881073
ZKSCAN8	-0.398899727	0.002819373	0.368049568
SPAG4	0.500456146	0.003096628	0.379134431
LMTK3	0.52192865	0.004630231	0.423806794
PANX2	0.543312666	0.004685004	0.423806794
RNF43	0.583175321	0.008196349	0.52972599
PLEKHH1	-0.332308377	0.008291401	0.53120832
SRMS	0.55637897	0.009492441	0.551961665
DSG2	-0.525819006	0.010146077	0.554466948
MROH6	0.522433855	0.010514687	0.554466948
SORBS2	-0.532715794	0.012983838	0.586198134
MAPK8IP2	0.650025745	0.013719146	0.586198134
TMEM64	-0.649116541	0.015978754	0.598808379
USP34	-0.358699016	0.020648921	0.632133347
SLC28A3	-0.555051029	0.020701175	0.632133347
TAOK1	-0.348745059	0.02099021	0.632473972
ZNF107	-0.314908986	0.033812255	0.698332359
NAPRT	0.500739412	0.038257395	0.712030093
LCN12	-0.324723838	0.046572045	0.733249327

**Table 2:** Differentially expressed and metastasis associated miRNAs in ceRNA network.

	logFC	P Value	adj.P.Val
hsa-miR-4529-5p	0.083182	0.001964317	0.423677628
hsa-miR-4732-5p	0.164886	0.002305116	0.423677628
hsa-miR-205-5p	-1.62982	0.00355452	0.526305888
hsa-miR-5691	-0.64002	0.005250125	0.647807062
hsa-miR-4759	0.243061	0.007832781	0.82710765
hsa-miR-767-5p	1.296216	0.010659283	0.910548747
hsa-miR-203b-3p	-1.32773	0.012570775	0.982582917
hsa-miR-4435	0.260738	0.013897397	0.982582917
hsa-miR-92a-1-5p	-0.71087	0.014958938	0.982582917
hsa-miR-3156-3p	0.496469	0.015760554	0.982582917
hsa-miR-105-5p	1.46322	0.017023377	0.982582917
hsa-miR-196a-3p	0.704418	0.018169254	0.982582917
hsa-miR-1538	-0.37858	0.020428508	0.982582917
hsa-miR-769-3p	-0.55012	0.037614062	0.982582917
hsa-miR-6783-3p	-0.41037	0.038042665	0.982582917
hsa-miR-503-5p	0.67017	0.038709206	0.982582917
hsa-miR-155-3p	-0.53033	0.040324782	0.982582917
hsa-miR-6820-3p	-0.41182	0.042252905	0.982582917
hsa-miR-6802-3p	-0.39398	0.046795738	0.982582917

**Table 3:** Differentially expressed and metastasis associated lncRNAs in ceRNA network.

	logFC	P Value	adj. P.Val
LINC01742	0.118378	1.14E-06	0.000521506
MIR583HG	0.11855	0.000330806	0.06510711
LINC01920	0.012082	0.001824527	0.251284985
LINC02080	0.032763	0.006593197	0.603747219
ZNF197-AS1	-0.104	0.006655724	0.603747219
LINC00028	0.086792	0.006668459	0.603747219
TESC-AS1	0.012969	0.010453491	0.777691137
FAM66E	0.010742	0.016804334	0.991905035
CATIP-AS2	0.0925	0.019484229	1
CYP4A22-AS1	-0.12903	0.019917265	1
TPM1-AS	-0.12722	0.031181472	1
GPR50-AS1	0.002362	0.032409713	1
ACVR2B-AS1	-0.18608	0.033049232	1
N4BP2L2-IT2	-0.10386	0.038352681	1
SIX3-AS1	0.16012	0.044972065	1
LINC00407	0.027636	0.045427248	1

### Identification of the Prognostic Biomarkers

**Figure 4:**
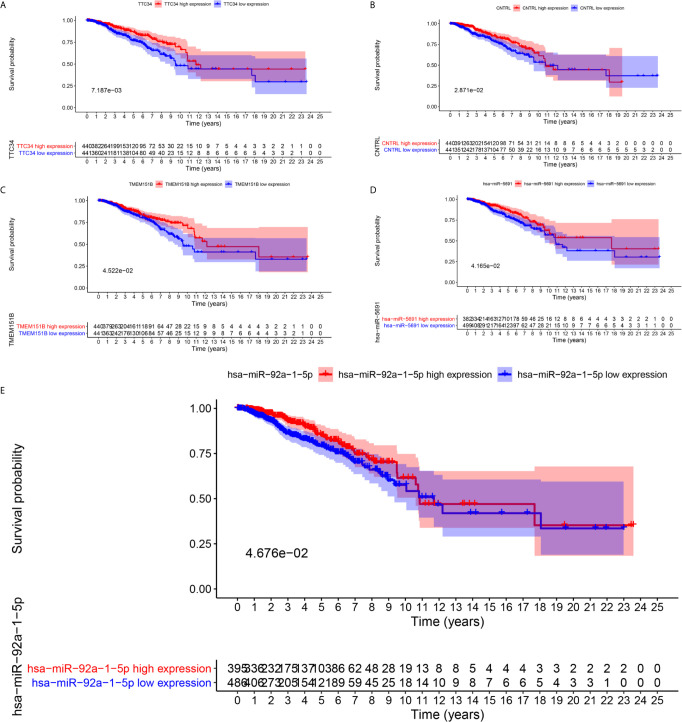
Kaplan–Meier survival curves of whose higher expression showed the higher OS. TTC34 **(A)**, CNTRL **(B)**, TMEM151B **(C)**, hsa-miR-5691 **(D)**, hsa-miR-92a-1-5p **(E)**.

**Figure 5:**
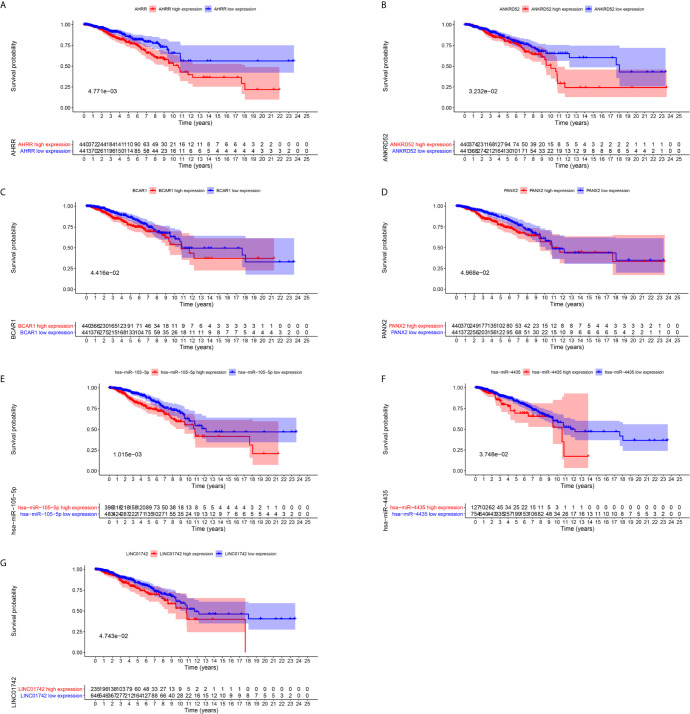
Kaplan–Meier survival curves prognostic. RNAs whose higher expression showed the lower OS. AHRR **(A)**, ANKRD52 **(B)**, BCAR1 **(C)**, PANX2 **(D)**, hsa-miR-105-5p **(E)**, hsa-miR-4435 **(F)**, LINC01742 **(G)**.

To further explore the prognostic significance of RNAs in metastasis associated ceRNA, K-M survival curves were plotted to observe the correlation between the expression levels of RNAs and the OS of breast cancer patients. The results of K-M survival curves and log rank test suggested that the expression dysregulation of 7 mRNAs (AHRR, TTC34, CNTRL, ANKRD52, BCAR1, TMEM151B and PANX2), 4 miRNAs (hsa-miR-105-5p, hsa-miR-4435, hsa-miR-5691 and hsa-miR-92a-1-5p) and lncRNA LINC01742 in metastasis associated ceRNA were significantly related to the OS of breast cancer patients (**Supplementary Tables S11**–**S13**). As shown in **Figure 4**, breast cancer patients with higher expression level of TTC34 (**Figure 4A**), CNTRL (**Figure 4B**), TMEM151B (**Figure 4C**), hsa-miR-5691 (**Figure 4D**), and hsa-miR-92a-1-5p (**Figure 4E**) showed the higher OS. Inversely, breast cancer patients with higher expression level of AHRR (**Figure 5A**), ANKRD52 (**Figure 5B**), BCAR1 (**Figure 5C**), PANX2 (**Figure 5D**), hsa-miR-105-5p (**Figure 5E**), hsa-miR-4435 (**Figure 5F**), and LINC01742 (**Figure 5G**) showed the lower OS. The results suggested that metastasis associated RNAs, including AHRR, TTC34, CNTRL, ANKRD52, BCAR1, TMEM151B, PANX2, hsa-miR-105-5p, hsa-miR-4435, hsa-miR-5691, hsa-miR-92a-1-5p, and LINC01742 could serve as the prognostic biomarkers of breast cancer patients.

### Identification and Validation of the Reliably Metastatic Biomarkers

**Figure 6:**
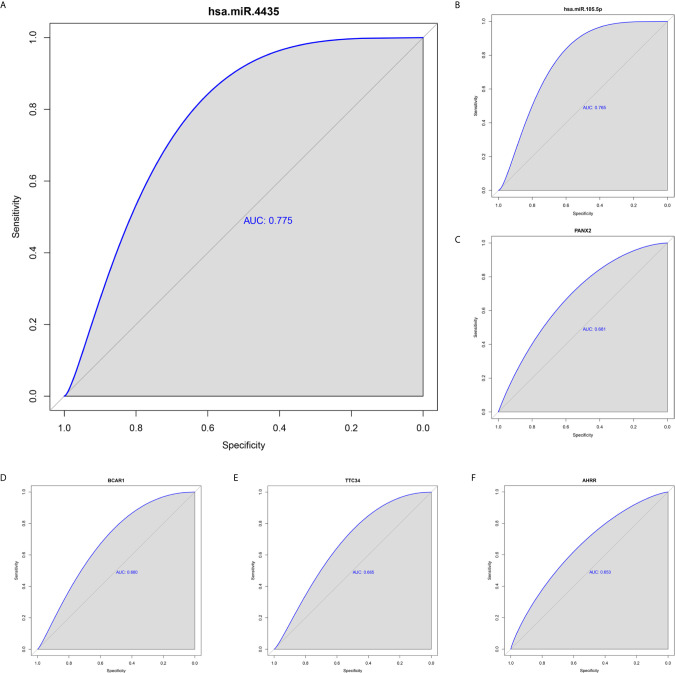
ROC analysis of 6 prognostic RNAs (only showing the AUC > 0.65). hsa-miR-4435 **(A)**, hsa-miR-105-5p (6), PANX2 **(C)**, BCAR1 **(D)**, TTC34 **(E)**, AHRR **(F)**. ROC, receiver operating characteristic curves.

**Figure 7:**
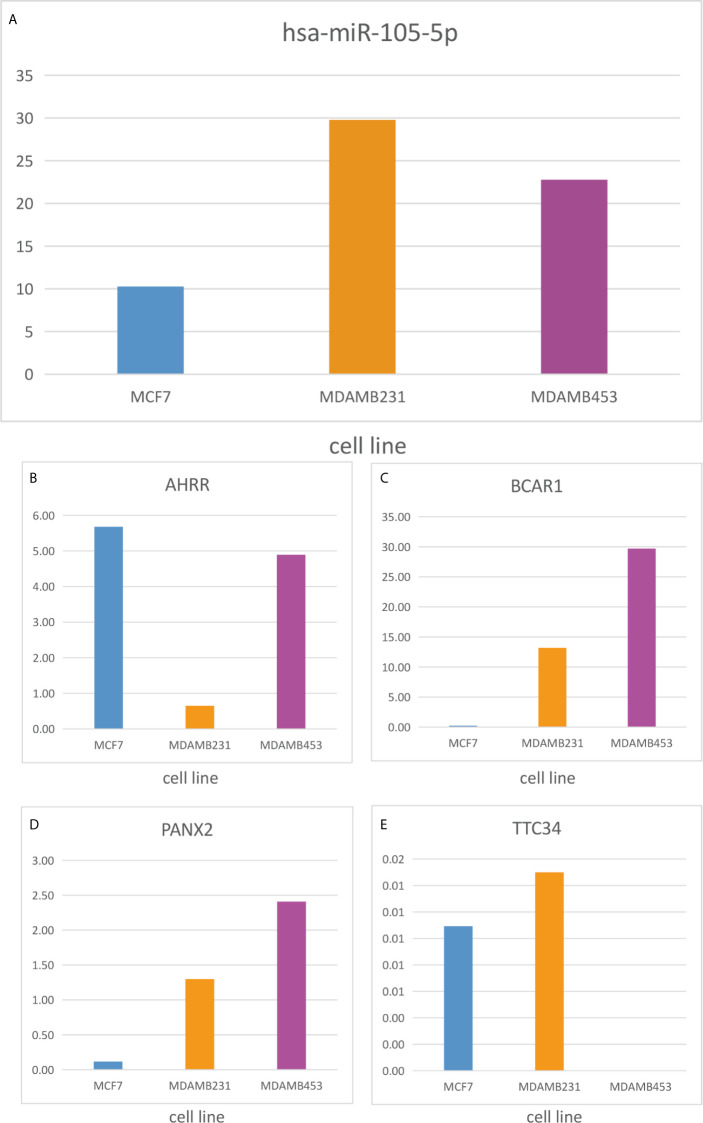
Expression level of the 5 RNAs between highly metastatic cell lines (MDAMB231, MDAMB453) and lowly metastatic cell lines (MCF7). hsa-miR-105-5p **(A)**, AHRR **(B)**, BCAR1 **(C)**, PANX2 **(D)**, TTC34 **(E)**.

**Figure 8:**
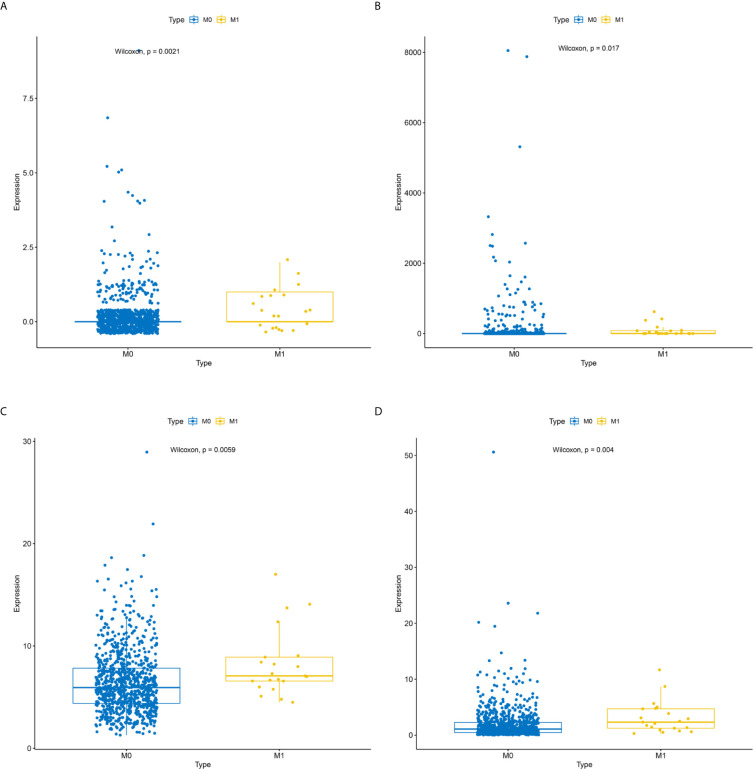
Expression levels of the 4 reliably metastatic biomarkers between M0 and M1 samples. hsa-miR-4435 **(A)**, hsa-miR-105-5p **(B)**, BCAR1 **(C)**, PANX2 **(D)**.

**Figure 9:**
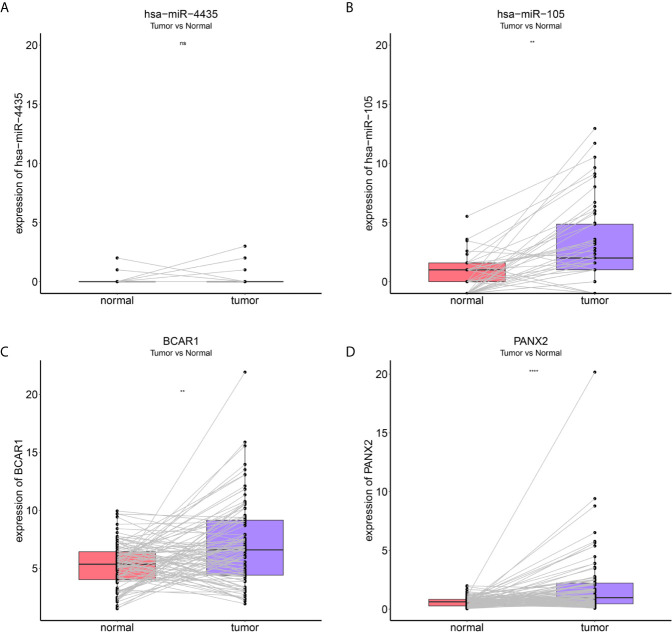
Expression levels of the 4 reliably metastatic biomarkers between 87 cancer tissues and 87 paired paracancerous tissues. hsa-miR-4435 **(A)**, hsa-miR-105-5p **(B)**, BCAR1 **(C)**, PANX2 **(D)**. **P < 0.01 and ****P < 0.0001, NS, not significant.

To identify metastatic biomarkers from the 12 prognostic and metastasis associated RNAs, the ROC analysis was carried out to investigate the accuracy of the RNAs for distinguishing the M1 samples from M0 samples. The results of ROC analysis suggested that hsa-miR-4435 (**Figure 6A**), hsa-miR-105-5p (**Figure 6B**), PANX2 (**Figure 6C**), BCAR1 (**Figure 6D**), TTC34 (**Figure 6E**), and AHRR (**Figure 6F**) were selected as the metastatic biomarkers because the AUC values of the 6 RNAs were greater than 0.65. In addition, for further verify the accuracy of 6 metastatic biomarkers, we compared the expression level of the 5 RNAs (hsa-miR-4435 was be excluded because of lacking of data) between high metastatic cell lines (MDA-MB-231 MDA-MB-453) and low metastatic cell lines (MCF-7).Obviously, only hsa-miR-105-5p, BCAR1 and PANX2 showed higher expression in highly metastatic cell lines (**Figures 7A, C, D**). Therefore, AHRR and TTC34 were removed from the biomarkers (**Figures 7B,E**). Furthermore, we also compared the expression levels of the 4 RNAs between M0 and M1 samples to elucidate the role of these 4 RNAs in metastasis of breast cancer. Surprisingly, all of them were upregulated in M1 samples compared to M0 sample (**Figure 8** **)**. On the other hand, we also compared the expression levels of these 4 RNAs between 87 cancer tissues and 87 paired paracancerous tissues. Notably, hsa-miR-4435 did not show the difference between 87 cancer tissues and 87 paired paracancerous tissues (**Figure 9A**) and only other three RNAs showed the difference (**Figures 9B–D** **)**. Thus, three reliably metastatic biomarkers (hsa-miR-105-5p, BCAR1 and PANX2) were reserved to differentiate M0 and M1 samples.

### Molecular Mechanism of Reliably Metastatic Biomarkers

**Figure 10:**
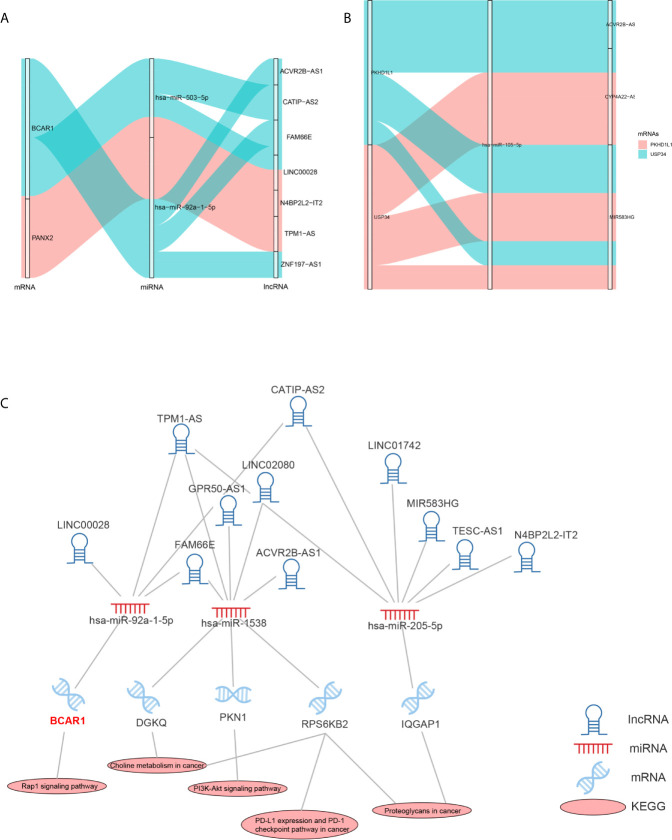
Regulatory mechanism of hsa-miR-105-5p, BCAR1 and PANX2. Sankey diagram shows the regulatory mechanism of BCAR1 and PANX2 **(A)**. Sankey diagram shows the regulatory mechanism of hsa-miR-105-5p **(B)**. Cancer associated KEGG pathways shows the regulatory mechanism of BCAR1 **(C)**.

As the DEMARs were markedly enriched in cancer related biological processes and pathways, it is most likely that 3 reliably metastatic biomarkers could influence the metastasis of breast cancer. Therefore, we further analyzed the regulatory mechanism of hsa-miR-105-5p, BCAR1 and PANX2. As shown in **Figure 10A**, lncRNA ACVR2B-ASQ, FAM66E, and ZNF197-AS1 could affect the expression of BCAR1 by a competitive combination with hsa-miR-92a-1-5p. CATIIP-AS2, FAMM66E, and LINC00028 could regulate the expression of BCAR1 by a competitive combination with hsa-miR-503-5p (**Figure 10A** **)**. Moreover, LINC00028, N4BP2L2-IT2, and TPM1-AS could regulate the expression of BCAR1 by a competitive combination with hsa-miR-503-5p and hsa-miR-92a-1-5p (**Figure 10A**). On the other hand, reliably metastatic biomarker hsa-miR-105-5p could regulate the expression of genes PKHD1L1 and USP34 (**Figure 10B**). In addition, hsa-miR-105-5p could be regulated by lncRNA CYP4A22-AS1 and MIR583HG.Furthermore, we also extracted the cancer associated KEGG pathways and the corresponding RNAs to show the regulatory mechanism of breast cancer. As shown in **Figure 10C**, the reliably metastatic biomarkers BCAR1 could regulate the Rap 1 signaling pathway (**Figure 10C**). However, hsa-miR-105-5p and PANX2 were not detected (**Figure 10C**).

## Discussion

Breast cancer, a common and malignant cancer in women, is one of the main causes for the cancer-related deaths (25). Metastasis of breast cancer is one of the causes of poor prognosis (26). Due to lacking specific diagnostic and prognostic biomarkers of metastatic breast cancer, patients have low survival rate. Therefore, it is urgent to explore potential mechanisms of metastasis and screen candidate biomarkers for monitoring the metastasis of breast cancer. Although researchers have revealed that the abnormal expression of ceRNAs plays a significant role in the occurrence, development and prognosis of tumors (15), few studies conducted on this aspect in metastatic breast cancer.

In the present research, the RNAs expression profiles of 899 primary breast cancer patients (including 878 M0 patients and 21 M1 patients) and 90 noncancerous tissue samples from TCGA were analyzed. Then two metastasis associated lncRNA-(up/down) miRNA-mRNA sub-networks were constructed and four metastatic biomarkers (BCAR1, PANX2, hsa-miR-105-5p) were identified from 12 prognostic biomarkers by bioinformatic analysis. Finally, combined with the KEGG signaling pathways, it could be speculated that the BCAR1 could affect the occurrence and metastasis of breast cancer by regulating the Rap 1 signaling pathway. In a word, the present study provided supporting data and theoretical basis for the study of ceRNAs in the metastasis of breast cancer.

BCAR1, whose full name is breast cancer antiestrogen resistance protein 1, encoding an adaptor/scaffold protein, has been reported to be involved in the regulation of signal transduction, actin cytoskeleton remodeling, and the stability of cell structure, especially as a mechanically sensitive regulator of filamentous pseudopod stability (27–30). In addition, BCAR1 has been shown to enhance tumor proliferation, invasion, and metastasis in several cancers (31–33). Furthermore, some oncogenes, such as ErbB2(erb-b2 receptor tyrosine kinase 2), PTEN (phosphatase and tensin homolog), and PI3KCA (phosphatidylinositol-4, 5-bisphosphate 3-kinase catalytic subunit alpha) promote tumor progression *via* the phosphorylation of BCAR1 (27). Notably, up-regulation of BCAR1 can alter the morphology of breast epithelial cell resulting in that the occurrence and development of breast cancer, which is exactly consistent with our result that the expression of BCAR1 was clearly upregulated in M1 patients compared with M0 samples (**Figure 8C**). Hence, BCAR1 might play a key role in the occurrence and development of tumor and could be used as a reliably metastatic biomarker (34, 35).

PANX2, whose full name is pannexin 2, encoding the protein pannexin 2, belonging to the innexin family, is the structural components of gap junctions (36). It has been demonstrated by previous studies that PANX2 is abundantly expressed in the central neuronal system, and its main function is participating in neuronal development and adult neurogenesis (37, 38). Moreover, the abnormal expression of PANX2 was associated with the occurrence the development and progression of certain diseases, like neoplasms, multiple sclerosis, migraines, hypertension and so on (39). In recent research, Fish et al. demonstrated that oncRNA (orphan noncoding RNAs), which they had named T3p, can promote tumor metastasis by acting as an inhibitor of RISC complex and increasing the expression of NUPR1 and PANX2 (40). Furthermore, Kim et al. also found that PANX2 might be used as a novel prognostic indicator for CCRCC patients. Nevertheless, few studies concentrated on the regulating mechanism of PANX2 (41). Thus, more studies were urgent to elucidate the regulating mechanism of PANX2.

Currently, as for the other metastatic biomarkers hsa-miR-105-5p, it remains unknown that it is associated with the development of breast cancer. Besides, a few researches investigated that they participated in the occurrence and progression of other tumors. Fang et al. suggested that the expression level of hsa-miR-105-5p was related to the survival of HCC patients (42).

In conclusion, the present study identified 12 biomarkers that might be involved in the prognosis of breast cancer by constructing a ceRNAs network. What’s more, the four of them were selected as the reliably metastatic biomarkers by ROC and expression analysis. In a word, these results suggested that BCAR1, PANX2, hsa-miR-105-5p might play a vital role in the progression of breast cancer and might be served as novel prognostic indicators for breast cancer, which might provide a new perspective for metastasis of breast cancer and contributed to the treatment of breast cancer. However, the regulating mechanisms of them were unclear and needed to further study to understand their roles in the metastasis of breast cancer.

## Data Availability Statement

The original contributions presented in the study are included in the article/**Supplementary Material**. Further inquiries can be directed to the corresponding author.

## Author Contributions

DQ and XM designed research. DQ, QZ, and XM carried out research. DQ, YQ, and DW analyzed data. DQ, BY, TZ, and JQ drawn the figures and made the tables. DQ and XM wrote the manuscript. All authors contributed to the article and approved the submitted version.

## Conflict of Interest

The authors declare that the research was conducted in the absence of any commercial or financial relationships that could be construed as a potential conflict of interest.

## Supplementary Material

Supplementary Figure 1 Under the cutoff of P-value < 0.05, differentially expressed RNAs between breast cancer patients and noncancerous tissue samples **(A)**, and between M0 and M1 samples **(B)**.

Supplementary Figure 2 Biological function of differentially expressed and metastasis associated lncRNAs.

Supplementary Table 1 Differentially expressed mRNAs between breast cancer patients and noncancerous tissue samples.

Supplementary Table 2 Differentially expressed miRNAs between breast cancer patients and noncancerous tissue samples.

Supplementary Table 3 Differentially expressed lncRNAs between breast cancer patients and noncancerous tissue samples.

Supplementary Table 4 Differentially expressed mRNAs between M0 and M1 samples.

Supplementary Table 5 Differentially expressed miRNAs between M0 and M1 samples.

Supplementary Table 6 Differentially expressed lncRNAs between M0 and M1 samples.

Supplementary Table 7 The results of KEGG pathway analysis of differentially expressed and metastasis associated mRNAs.

Supplementary Table 8 The results of GO analysis of differentially expressed and metastasis associated mRNAs.

Supplementary Table 9 Biological function of differentially expressed and metastasis miRNAs.

Supplementary Table 10 Biological function of differentially expressed and metastasis lncRNAs.

Supplementary Table 11 The results of K-M survival curves and log rank test of mRNAs in ceRNA networks which related to the OS of breast cancer patients.

Supplementary Table 12 The results of K-M survival curves and log rank test of miRNAs in ceRNA networks which related to the OS of breast cancer patients.

Supplementary Table 13 The results of K-M survival curves and log rank test of lncRNAs in ceRNA networks which related to the OS of breast cancer patients.
