# Supplementary figures and images for: Integrated Analysis of ceRNA Network Reveals Prognostic and Metastasis Associated Biomarkers in Breast Cancer
Source: Front Oncol. 2021 May 13;11:670138. doi: 10.3389/fonc.2021.670138 (PMC8158160; doi:10.3389/fonc.2021.670138)

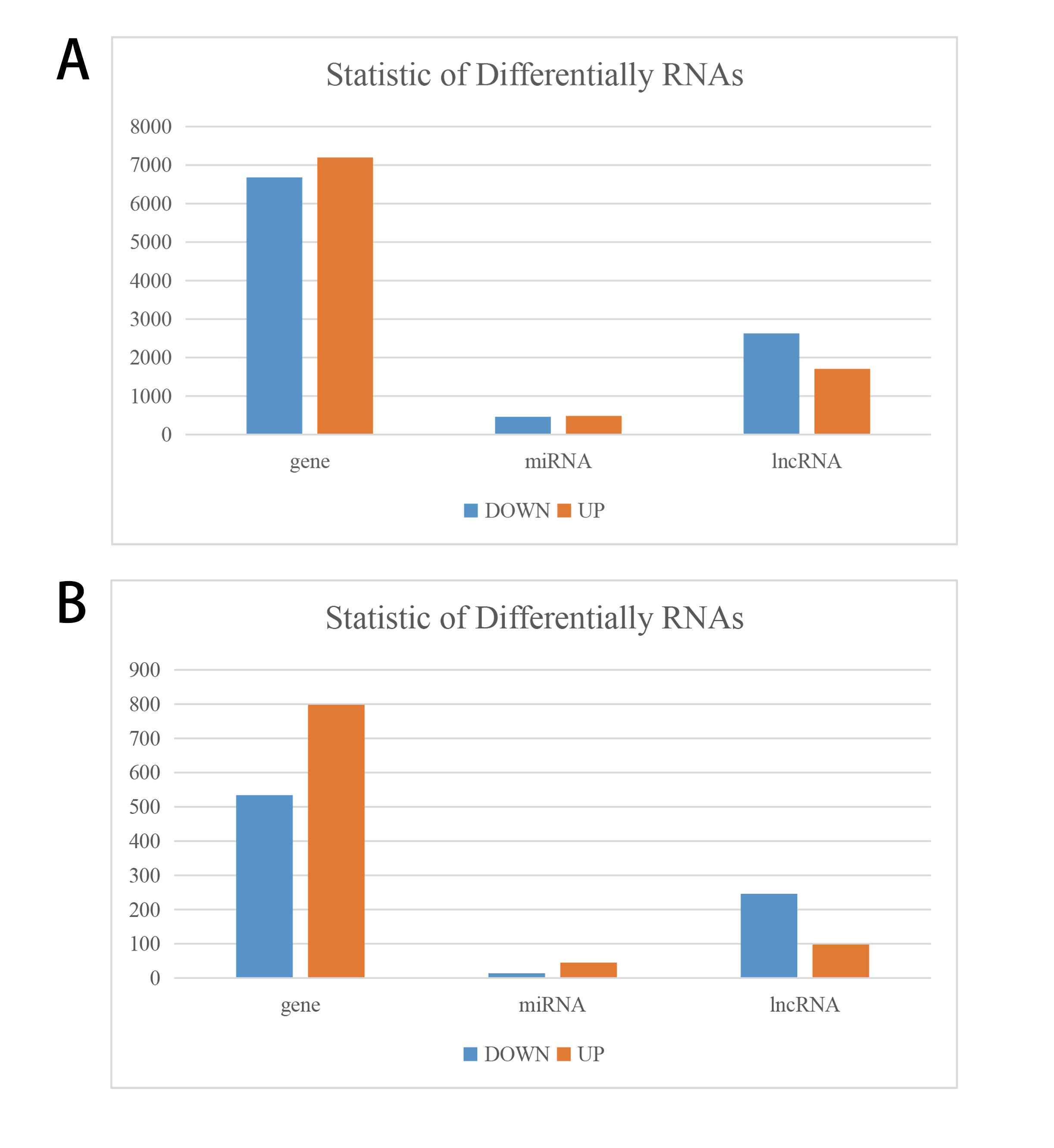

Supplement: Supplementary Figure 1 — Under the cutoff of P-value < 0.05, differentially expressed RNAs between breast cancer patients and noncancerous tissue samples (A), and between M0 and M1 samples (B). [file Image_1.tif]

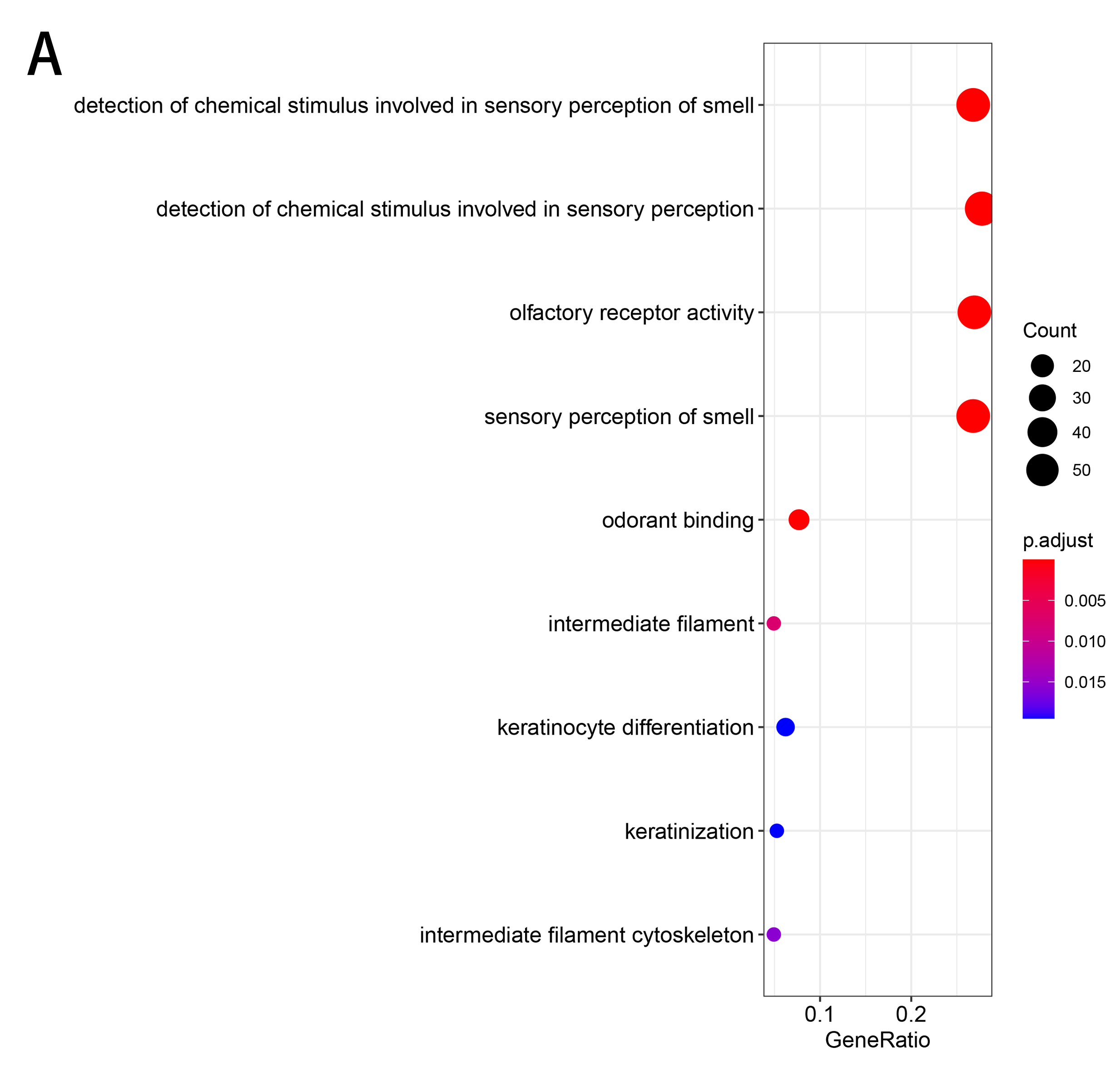

Supplement: Supplementary Figure 2 — Biological function of differentially expressed and metastasis associated lncRNAs. [file Image_2.tif]
